# The Human Homolog of *Escherichia coli* Endonuclease V Is a Nucleolar Protein with Affinity for Branched DNA Structures

**DOI:** 10.1371/journal.pone.0047466

**Published:** 2012-11-05

**Authors:** Cathrine Fladeby, Erik Sebastian Vik, Jon K. Laerdahl, Christine Gran Neurauter, Julie E. Heggelund, Eirik Thorgaard, Pernille Strøm-Andersen, Magnar Bjørås, Bjørn Dalhus, Ingrun Alseth

**Affiliations:** 1 Department of Microbiology, Oslo University Hospital HF and University of Oslo, Rikshospitalet, Oslo, Norway; 2 Department of Medical Biochemistry, Oslo University Hospital HF and University of Oslo, Rikshospitalet, Oslo, Norway; 3 Centre for Molecular Biology and Neuroscience (CMBN), Oslo University Hospital HF and University of Oslo, Rikshospitalet, Oslo, Norway; The University of Hong Kong, Hong Kong

## Abstract

Loss of amino groups from adenines in DNA results in the formation of hypoxanthine (Hx) bases with miscoding properties. The primary enzyme in *Escherichia coli* for DNA repair initiation at deaminated adenine is endonuclease V (endoV), encoded by the *nfi* gene, which cleaves the second phosphodiester bond 3′ of an Hx lesion. Endonuclease V orthologs are widespread in nature and belong to a family of highly conserved proteins. Whereas prokaryotic endoV enzymes are well characterized, the function of the eukaryotic homologs remains obscure. Here we describe the human endoV ortholog and show with bioinformatics and experimental analysis that a large number of transcript variants exist for the human endonuclease V gene (*ENDOV*), many of which are unlikely to be translated into functional protein. Full-length ENDOV is encoded by 8 evolutionary conserved exons covering the core region of the enzyme, in addition to one or more 3′-exons encoding an unstructured and poorly conserved C-terminus. In contrast to the *E. coli* enzyme, we find recombinant ENDOV neither to incise nor bind Hx-containing DNA. While both enzymes have strong affinity for several branched DNA substrates, cleavage is observed only with *E. coli* endoV. We find that ENDOV is localized in the cytoplasm and nucleoli of human cells. As nucleoli harbor the rRNA genes, this may suggest a role for the protein in rRNA gene transactions such as DNA replication or RNA transcription.

## Introduction

The genomes of all organisms are constantly challenged by agents, produced inside the cell or in the environment, that cause damage to the DNA. DNA base damage may lead to errors in replication and transcription, compromising the integrity of the genome. Three of the four bases present in DNA (cytosine, adenine, and guanine) contain an exocyclic amino group. Loss of this group by deamination occurs spontaneously under physiological conditions via a hydrolytic reaction [1], [2]. This process is greatly enhanced by agents such as reactive oxygen radicals, UV radiation, heat, ionizing radiation, nitrous acid, nitric oxide, and sodium bisulfite [3]–[7].

It is estimated that a few hundred amino groups are lost from the DNA bases spontaneously in each cell every day, most frequently from cytosine bases. Adenine deamination occurs only at a rate of 2–3% compared to that of cytosine [8]. Deamination of cytosine and adenine produces uracil and hypoxanthine (Hx), respectively, both having miscoding properties. In addition, Hx in DNA might be the result of misincorporation of 2′-deoxyinosine triphosphate (dITP) during DNA replication [9]. In this case dITP is incorporated opposite cytosine and is also read as guanine by the DNA polymerases. Thus, at least in *Escherichia coli*, dITP incorporation is nonmutagenic [10].

Whereas uracil in DNA is removed by uracil DNA glycosylases [11], the principal enzyme for removal of Hx in *E. coli* is endonuclease five (endoV) encoded by the *nfi* gene [12]. This enzyme binds to and cleaves the second phosphodiester bond 3′ to Hx in an Mg^2+^ dependent manner generating 3′-OH and 5′-P termini [13], [14]. Endonuclease V does not on its own remove the damage from DNA and additional proteins are thus required to complete repair. This process is poorly understood but has been shown to be reconstituted with recombinant endoV, DNA polymerase I and DNA ligase [15]. *E. coli* cells lacking endoV have a normal spontaneous mutation frequency, however upon exposure to nitrous acid *nfi^−^* cells are mutators showing elevation in AT→GC and GC→AT transition as well as GC→CG transversion mutations [16]. *E. coli* endoV is a rather promiscuous enzyme acting on different substrates including uracil [17], [18], xanthine (deaminated guanine) [19], apurinic/apyrimidinic (AP) sites [14], urea residues [14], mismatches [20] and also structure substrates such as insertion and deletion loops, 5′-flaps, hairpins and pseudo-Y structures [21]. The ability of *E. coli* endoV to recognize all three deamination products in DNA is unique and is not shared by any of the other known repair enzymes. Finally, is has been shown that endoV from *Thermotoga maritima* (*Tma*) possesses both 5′ and 3′ exonuclease activities and a potential role for these activities in end-processing after Hx incision was suggested [22]. The 3-dimensional structure of *Tma* endoV in complex with Hx-containing DNA was recently determined [23]. The structure reveals the presence of a wedge motif (PYIP) involved in damage detection and DNA strand separation at the site of the lesion. The deaminated adenine lesion is rotated approximately 90° into a recognition pocket where it is tightly coordinated by hydrogen-bonding interactions.

Homologs of endoV are widespread in nature and are found in all three domains of life [23]. In addition to *E. coli*, endoV homologs have been characterized from *Archaeglobus fulgidus* [24], *T. maritima* [25], *Ferroplasma acidarmanus* (in fusion with *O* ^6^-alkylguanine-DNA alkyltransferase active site domain) [26] and *Salmonella typhimurium* [27], however knowledge about the eukaryotic counterparts is sparse. cDNA for endoV from mice has been cloned, however no robust enzyme activity for Hx or other tested substrates were found [28]. In this work we have characterized the human variant of endonuclease V by identification of isoforms, subcellular localization and biochemical assays.

## Materials and Methods

### Ethics statement

A commercially available tissue array was used and ethical principles maintained by the manufacturer (Origene) (http://www.origene.com/Tissue/Tissue_QC.aspx).

### Bioinformatics analysis

Protein and mRNA derived sequences were obtained from GenBank [29] and other NCBI database resources [30] and from the Ensembl project [31]. The large number of human transcript variants was also investigated in the Ensembl and the UCSC [32] genome browsers. Protein structural disorder was predicted with DISOPRED2 [33].

A multiple sequence alignment of human ENDOV and *Tma* endoV as well as 13 and 8 additional eukaryotic and bacterial homologs, respectively, was generated with Muscle [34]. An alignment of the human (target) and *Tma* (template) sequences based on this multiple sequence alignment was manually edited in order to move insertions and deletions out of secondary structure elements in the structural modeling template from Dalhus *et al.* [23] (Protein database (PDB) identifier 2W35). With this alignment, a model of human ENDOV was generated with SwissModel [35] employing standard homology modeling.

### Cell culture and transfection

Human embryonic (HE) fibroblasts were obtained from the National Institute of Public Health (Folkehelsa, Oslo, Norway) and cell lines HCT116 (human colon epithelial cells), ACHN (human kidney epithelial cells) and HeLa S3 (human cervix epithelial cells) were purchased from American Type Culture Collection (ATCC). HE cells were cultured in a 1∶1 mix of minimal essential medium (MEM; Gibco, Life Technologies, Carlsbad, CA, USA) and Dulbecco's modified Eagle's medium (DMEM; Gibco) supplemented with 10% fetal bovine serum (Standard quality FBS, PAA lab, Austria), 1× GlutaMAX (200 mM, Gibco), and 1× penicillin-streptomycin (10000 U/ml, Lonza, Basel, Switzerland). HCT116, ACHT and HeLa cells were cultured in DMEM supplemented with 10% FBS, 1× GlutaMAX, and 1× penicillin-streptomycin. Transient transfections were performed with FUGENE (Invitrogen, Life Technologies), according to the supplied protocol.

### Cell cycle synchronization and analysis by flow cytometry

Synchronization of the cells in G0 phase was achieved by culturing cells as a confluent layer for 72 h followed by serum starvation (0.2% serum) for 72 h. The cells were released from G0 by trypsination (Trypsin-EDTA 200 mg/l, Lonza) for 4 min at 37°C and cultivated in standard growth medium at 25% confluence. Cells were harvested by trypsination at indicated time points, washed in ice-cold PBS and stored at −20°C. Cells used for phase analysis were resuspended in PBS and fixed by addition of ice-cold 100% ethanol to a final concentration of 70% and stored at −20°C. For FACS analysis, the cells (about 1 mill/ml) were stained with 50 µg/ml propidium iodide (Sigma-Aldrich, St.Louis, MO, USA) in 4 mM Na-citrate buffer containing 0.1 mg/ml RNaseA (Molzyme GmbH & Co, Bremen, Germany) and 0.1% Triton X-100 (Sigma-Aldrich) for 10 min at 37°C and put on ice. Cells were subjected to flow cytometry analysis (BD LSRII flow cytometer, Becton Dickinson, San Jose, California, USA) and the results were analysed with CellQuest software (Becton Dickinson).

### Total RNA isolation, cDNA synthesis and quantitative real-time RT-PCR

Total RNA was isolated from frozen cell pellets using RNeasy kit (QIAGEN, Hilden, Germany) according to the manufacturer's instructions. cDNA was generated from total RNA samples using the High-capacity cDNA reverse transcription kit (Applied Biosystem, Life Technologies). Human *ENDOV* mRNA levels was determined with primers amplifying exons 2 to 3 or exons 6 to 8 (Table S1; Eurofins MWG Operon, Ebersberg, Germany) using the Power SYBR Green PCR master mix and the Step One Plus Real-Time PCR system (Applied Biosystem) according to the kit and system instructions. All samples were run in triplicate, and melting point analyses were performed to confirm the specificity of the PCR reaction. *GAPDH* (Table S1, primers 5 and 6) was used as the reference gene for normalization, and G0 as the reference sample for RQ calculation.

For measurement of human *ENDOV* expression in normal and cancer tissue, TissueScan™ Cancer Survey cDNA Arrays (CSRT303; OriGene Technologies, Rockville, USA) were used. These arrays consisted of cDNA prepared from pathologist-verified human tumor tissue obtained from 18 different tissues normalised against β*-ACTIN*. Primers covering exons 6 to 8 were used (Table S1). Additional clinical information for each sample can be found at http://www.origene.com/qPCR/Tissue-qPCR-Arrays. Results are shown for 13 of the tissues, whereas 5 were omitted due to high uncertainty in the values obtained.

### Nothern Blot analysis

Total RNA was isolated from HE cells using TRIzol Reagent (Ambion, Applied Biosystems) according to manufacturer's instructions. mRNA was isolated from total RNA using the MicroPoly(A)Purist Kit (Ambion) and 5 µg/lane was subjected to 1% denaturing agarose gel electrophoresis at 5 V/cm. mRNA was transferred to an BrightStar-Plus membrane (Ambion) by downward transfer from gel and crosslinked to the membrane at 120 mJ/cm^2^ in a CL-1000 UV-Crosslinker (UVP, Upland, California, USA). The Northern Max kit (Ambion) was used in the blotting, with prehybridization/hybridization and washing steps performed as described by the manufacturer. Primers for PCR amplification of the probes are listed in Table S1. The β -*ACTIN* cDNA probe was from Clontech (Takara Bio, Otsu, Japan). The probes (178 bp for exon 3, 378 bp for exons 4–8 and 342 bp for exon 10) were labeled using Rediprime II Random Prime labeling system (Amersham Biosciences, GE Healthcare Bio-Sciences AB, Uppsala, Sweden) and EasyTide dCTP (α-^32^P) (PerkinElmer, Massachusetts, USA). Hybridization signals were detected and quantified by phosphorimaging (Typhoon 9410) and ImageQuant TL software (Molecular Dynamics, California, USA). The amount of *ENDOV* transcripts was calculated relative to β-*ACTIN*.

### Immunofluorescence microscopy

Human *ENDOV* (exons 1–10) was subcloned into pEGFP N- and C-vectors (Clontech) by Genescript (Piscataway, New Jersey, USA). HeLa cells were grown to 60–70% confluence on 2-well chamber slides and transfected with the different pEGFP constructs using the FuGENE6 (Roche, Mannheim, Germany) reagent according to the manufacturer's instructions. Cells were fixed for 15 min in PBS containing 4% paraformaldehyde, washed and quenched in 20 mM glycine in PBS for 10 min. Permeabilization was performed in 0.1% Triton X-100 in PBS for 10 min followed by blocking with 10% FBS in PBS for 30 min. All labelling steps were carried out in the blocking buffer. Cells were incubated with monoclonal primary antibodies against fibrillarin (Abcam, Ab4566) for 1 h, washed, and further incubated for 1 h with Alexa 595–conjugated anti-mouse antibodies (Molecular Probes Europe, Life Technologies). Cells were then washed in PBS and coverslips were mounted with Mowiol (Sigma-Aldrich). Confocal images were acquired with Carl Zeiss LSM 510 CLSM laser scanning microscope (Jena, Germany).

Protein extracts for western analysis were made by adding ice-cold RIPA lysis buffer containing protease inhibitor cocktail (P8340, Sigma-Aldrich) to the transfected cells. Cells were collected, sonicated for 2×20 sec and spun down at 12.000 *g* for 10 min. 30 µg cell extract were boiled with NuPAGE LDS sample buffer (Invitrogen) and subjected to SDS-PAGE using 10% NuPAGE gels (Invitrogen). Proteins were transferred to PVDF membrane using iBlot® Gel Transfer Device (Life Technologies), detected by incubating the membrane ON at 4°C with anti-GFP antibody (Abcam, Ab290) followed by incubation for 1 h at RT with HRP-conjugated secondary antibody and visualised by Immun-Star WesternC chemiluminescence kit (BioRad Laboratories, California, USA) using the ChemiDoc MP System (BioRad).

### Design of constructs for recombinant protein expression

The nucleotide sequence for the human *ENDOV* transcript (exons 1–9, 309 residues) was synthesised by Genscript Inc. (Piscataway, New Jersey, USA) with optimal codon usage for expression in *E. coli* in the pET28b vector (Novagen, Darmstadt Germany) using the NdeI and EcoRI restriction sites. No protein was produced from this construct upon induction of *E. coli* BL21-CodonPlus(DE3)-RIL cells (Stratagene, Agilent technologies, California, USA) and an N-terminal maltose binding protein (MBP)-ENDOV fusion construct was made as follows: The codon optimised sequence was amplified by PCR using primers 5′-ATATCCATGGCACTGGAAGCCGCCGGC-3′ and 5′-ATATGGATCCTTACTGCCAATCTTTACCCGCCTGTTCC-3′ for subcloning into the vector pETM-41 (EMBL, Heidelberg, Germany) using NcoI (underlined) and BamHI (underlined) to give a construct with ENDOV fused to an N-terminal MBP tag separated by a tobacco etch virus (TEV) protease cleavage site (pETM-41-MBP-TEV-ENDOV-Exon9). The fusion protein also contained an N-terminal hexahistidine tag in front of the MBP protein. This construct was further used as a template to design the corresponding fusion between MBP and the shorter isoform of human ENDOV (exon 1–10, 282 residues) by site-specific mutagenesis using the forward primer 5′-GGCGATTCTGGTGAAAGCTCTGCGCTGTGTTAGCCGCCGCAGGATCACTCTCCG-3′ together with its corresponding reverse and complementary oligonucleotide. A total of 6 nucleotide mutations in the underlined region transformed the amino acid sequence for ENDOV exon 9 from 280…GEGQ…283 to 280…ALC-Stop…. to give pETM-41-MBP-TEV-ENDOV-Exon10. The mutants ENDOV-RK, ENDOV-Y91A and ENDOV-Wg were designed by site-specific mutagenesis using the forward primers 5′- GCAGTCGTGAACATATCGACGATAGCCTGGGTCTGCCGGGTCC-3′, 5′- CTGACGGCGCCGGCGGTTAGCGGCTTTC-3′ and 5′- CGTATGGTGAGCCTGACGGCGGGGGGCGGTGGCGGCTTTCTGGCCTTCCGTGAAGTGCC-3′ with their corresponding reverse primers, respectively.

### Protein expression and purification

The pETM-41 based human ENDOV exon 10 WT and mutant constructs were transformed into chemically competent *E. coli* BL21-Codon Plus (DE3)-RIPL cells. The cells were grown in LB medium supplemented with 100 mg/l kanamycin at 37°C with shaking until the OD_600 nm_ reached ∼0.8. The temperature was lowered to 18°C before induction of the protein expression by 0.25 mM isopropyl β-d-1-thiogalactopyranoside (IPTG). After overnight expression at 18°C, cells were harvested by centrifugation and the cell pellets were resuspended in buffer A, containing 50 mM Tris pH 8.0, 300 mM NaCl, 10 mM imidazole and 10 mM β-mercaptoethanol (β-ME). Cells were lysed by ultrasonication (3×30 sec) followed by centrifugation for 30 min at 27000 *g* at 4°C. The recombinant His-MBP-ENDOV fusion proteins were extracted from the supernatant by Ni-NTA affinity chromatography, using 50 mM and 300 mM imidazole versions of buffer A for elution. Fractions rich in ENDOV were pooled, concentrated and dialysed at 4°C against a TEV buffer (50 mM Tris pH 8.0, 0.5 mM EDTA, 1 mM DTT). TEV protease with an N-terminal hexahistidine tag produced from a pSC563 plasmid (Courtesy of Prof. M Ehrmann, Cardiff University, UK) was added to the fusion proteins in ratio 1∶100 and incubated at 12°C overnight. After proteolysis, the protein mixtures were dialysed against buffer A, and the free His-MBP and TEV proteins were separated from ENDOV by a second Ni-NTA purification step. The untagged ENDOV proteins were collected in the flow-through and wash fractions, concentrated and applied to a Superdex 75 size-exclusion chromatography column (GE Healtcare) equilibrated with 50 mM NaCl, 50 mM Tris pH 8.0 and 10 mM β-ME. The purified human ENDOV was concentrated and stored at 4°C.

### Oligonucleotides and ^32^P labeling

The DNA substrates were made by combining the oligonucleotides (Eurofins MGW Operon; Table S2) in the following way (asterisk indicates the ^32^P labeled oligonucleotides): undamaged DNA: 1+2*, double stranded with hypoxanthine: 1+3*, double stranded with uracil: 4*+5, loop: 6*+7 hairpin: 8*+9, 3′-flap: 10+11+14*, 5′-flap: 10+12+13*, 3-way: 10*+13+14, pseudo-Y: 10*+13, fork: 10+11+14*+15 and Holliday junction: 16+17*+18+19. The DNA substrates were 5′ end labeled using T4 polynucleotide kinase (New England BioLabs, Hitchin, UK) in the presence of [γ-^32^P]ATP (Amersham Biosciences). Radioactive labeled oligonucleotides were annealed to their respective complementary strands by heating the solution to 90°C for 2 min and slowly cooling to room temperature. The DNA substrates were separated by 10% native PAGE, excised from the gel, eluted by diffusion in H_2_O and stored at 4°C.

### DNA nicking activity

Various amounts of ENDOV were mixed with 10 fmol substrate DNA, 5 ng pQE31 competitor DNA (Qiagen, QIAGEN, Hilden, Germany) and reaction buffer (10 mM Tris-HCl pH 7.5, 2 mM MgCl_2_, 50 mM KCl, 5% glycerol and 1 mM DTT) in a total volume of 10 µl, and incubated at 37°C for 30 min. The reactions were stopped by adding formamide loading buffer (90% formamide, 0.1% xylene cyanol, 0.1% bromphenol blue) and heating to 85°C for 3 min. Cleavage products were analysed by 20% PAGE (Long Ranger, 7 M urea, 1× taurin), visualised by phosphorimaging and quantified by ImageQuant TL.

### Electrophoretic mobility shift assay

The affinity of human ENDOV for damaged DNA was analysed by electrophoretic mobility shift assay (EMSA). Enzyme (amounts given in figure legends), 10 fmol DNA substrates and 5 ng pQE31 competitor DNA were incubated in a 10 µl reaction volume (5 mM CaCl_2_, 10 mM HEPES-KOH pH 7.4, 1 mM DTT and 20% glycerol) at 4°C for 15 minutes. DNA loading buffer (Fermentas) were added and the samples were separated by 10% native PAGE (Long Ranger, 1× taurin, 5 mM CaCl_2_) on ice. Results were visualised by phosphorimaging and quantified with ImageQuant TL software.

## Results

### Bioinformatics analysis of ENDOV demonstrates numerous splice variants

The endonuclease V gene has a scattered distribution in eukaryotes, most compatible with independent gene loss in multiple lineages. Publicly available sequence information shows that *ENDOV* orthologs are present in most plants and green algae, in echinoderms (*Strongylocentrotus purpuratus*) and in all three subphyla of the chordates, the vertebrates, tunicates, and cephalochordates. It is also found in sponges (*Amphimedon queenslandica*), cnidarians (*e.g. Nematostella vectensis*), and amoebozoans such as *Entamoeba histolytica* and *Dictyostelium discoideum*. Among the arthropods the gene appears to be completely missing in the largest group, the insects, but it is found in the genomes of at least some crustaceans (*e.g. Daphnia pulex* and *Caligus rogercresseyi*). Within the fungi, an *ENDOV* ortholog is found in the genome of *Schizosaccharomyces pombe* and the other three sequenced *Schizosaccharomyces* species, but is otherwise nearly, or possibly completely, absent. Endonuclease V also appears to be missing in apicomplexan protozoans such as *Plasmodium falciparum* and *Cryptosporidium parvum*. In summary, the ENDOV sequence is highly conserved in eukaryotes, but the gene itself appears to have been lost in a large fraction of the eukaryotic species.

**Figure 1:**
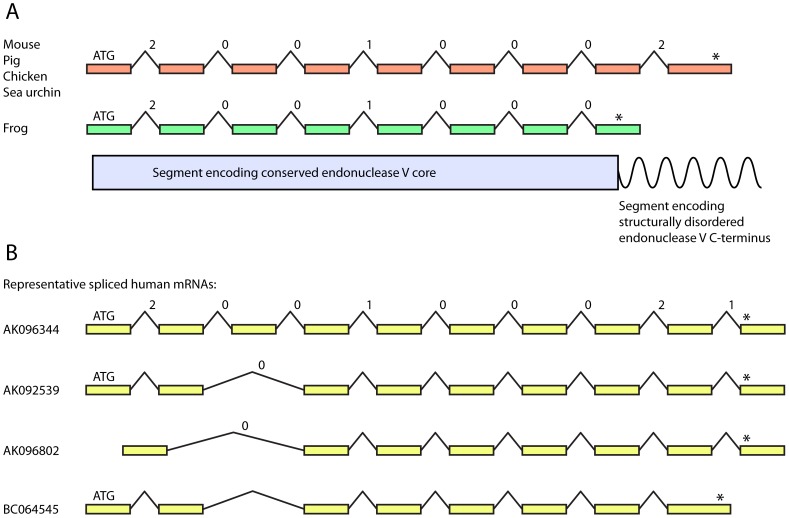
Schematic representation of splicing of human, other tetrapod and sea urchin *ENDOV* mRNAs. (**A**) The segments of the *ENDOV* transcripts encoding the conserved protein core are spliced identically, including the intron phases, in rodents, pig, chicken, frog and the echinoderm sea urchin. The position of the start (ATG) and stop (asterisk) codons are indicated above the relevant exons (not shown to scale), while intron phases are shown above the introns. These are defined as the position of the intron within a codon, with phase 0, 1, and 2 placed before the first nucleotide, after the first nucleotide, and after the second nucleotide, respectively. The structurally disordered and poorly conserved C-terminus of ENDOV is encoded by one or more 3′ exons and has variable length, *e.g.* ∼85 and ∼5 residues in mouse and frog, respectively. (**B**) Most previously published spliced human mRNAs are lacking one or several 5′ exons, in particular exon 3, while alternative splicing at the 3′ end results in multiple variants. Four representative full length mRNAs are shown with accession numbers from GenBank [29].

The mouse endonuclease V variant described by Moe *et al.* [28] comprises nine exons and translates into a protein with 338 residues (validated RefSeq [36] record NP_001158108). At least 20 publicly available expressed sequence tag (EST) sequences, representing processed mRNA from various tissues and life stages, confirm that this is the major splice variant for EndoV in mice (Figure 1A). The chicken genome [37] encodes an ENDOV ortholog that is spliced identically as in mouse, while the frog *Xenopus tropicalis* [38] ortholog is spliced identically for the seven 5′ introns (Figure 1A). Also for chicken and frog, this splicing pattern is supported by a number of published EST sequences. Frog ENDOV has a shorter C-terminus due to a stop codon in exon 8. The correctness of this stop codon is supported by both the genomic sequence and all ten available EST sequences spanning the 3′ region of the transcript. The pattern of splicing of exons 1–8 described above is conserved, also including the intron phases, in ENDOV orthologs in other mammals such as pig and rat, and in the more distantly related sea urchin *S. purpuratus* (Figure 1A). All protein sequences are listed in Figure S1.

The conserved splicing pattern results in metazoan endonuclease V proteins with a high degree of similarity with prokaryotic endoV orthologs. For example, the core region of mouse ENDOV, *i.e.* residues 1–250, encoded by exons 1–8 (Figure 1), aligns with full-length *E. coli* (223 residues) and *T. maritima* (225 residues) endoV with ∼32% and ∼29% sequence identity, respectively. Given this degree of similarity, it is likely that the 3D structures of metazoan ENDOV enzymes are very similar to the bacterial homologs (See Ref. [23] as well as PDB identifiers 3HD0, 3GOC, and 3GA2). The C-terminal segment of the above bilaterian ENDOV homologs, *e.g.* residues 251–338 of mouse ENDOV, is predicted to be structurally disordered (Figure 1 and S1). This part of ENDOV also appears to be under no selective pressure (Table S3). The ratio of non-synonymous (*K*a) and synonymous (*K*s) substitution rates for pairwise comparisons of ENDOV segments from mouse, rat, and Chinese hamster gives an average value of *K*a/*K*s = 0.19 for the core region, showing, as expected, purifying selection (*K*a/*K*s<1). For the C-terminal segment, average *K*a/*K*s = 1.49, strongly suggesting that during evolution there is no selective pressure for conserving the sequence of the C-terminal tail (*i.e.* all mutations are equally tolerated).

Splicing of human *ENDOV* transcripts has not previously been analysed in detail, but judging from the sequence data available in public databases there is a high degree of alternative splicing of this gene, resulting in a multitude of isoforms. The human protein with RefSeq identifier NP_775898 corresponds to an mRNA where exons 1–9 are spliced in the same fashion as in other bilateria (see above), but with an additional 3′ exon (Figure 1B). This transcript consists of 2858 nucleotides (nt) and will in the following be referred to as full-length (1–10). However, only one single (AK096344) of nine human *ENDOV* mRNAs available in GenBank is spliced in a similar fashion, while the remaining eight mRNAs all are lacking exon 3. There are more than 70 human ESTs, representing mRNAs from various tissues, available for *ENDOV*, but among the ∼30 ESTs comprising exons 1, 2 and 4, only 9 contains exon 3. Thus, the major fraction of available spliced human *ENDOV* transcript sequences is lacking exon 3. It is, however, highly unlikely that these transcripts will be translated into a folded and functional protein, since exon 3 builds a large part of the ENDOV core domain and contains residues involved in damage recognition, DNA strand cleavage, as well as the DNA strand separating PYIP motif [23]. While many of the human *ENDOV* transcripts comprise exons 5–8, splicing at the 3′ end results in more than eight alternative isoforms with less than ten of nearly fifty transcripts from the 3′ end of the gene having identical splicing as in mouse and chicken (Figure 1). None of the alternative 3′ region splice variants appears to be evolutionary conserved or to encode a structured protein domain. In conclusion, the majority of human *ENDOV* transcripts available in public databases are unlikely to produce functional protein. The C-terminal tail of ENDOV is not evolutionary conserved in metazoa and is found in many variants due to alternative, seemingly random, splicing in human cells.

### Characterization of *ENDOV* transcripts in human cells

As the sequence databases revealed a multitude of human *ENDOV* transcript variants, we aimed to verify their presence experimentally. Initially we tried to detect full-length *ENDOV* cDNA from different human cell types. Total RNA was purified from primary human fibroblasts, kidney and colon cell lines and subjected to cDNA synthesis and PCR analysis using exon 1 and exon 8 or 9-specific primers. With these primer sets we only amplified *ENDOV* transcripts lacking exon 3 (data not shown). However exon 3 containing transcripts were detected in these cells using exon 3 specific primers (data not shown). Based on these observations we assumed that full-length transcripts containing exon 3 most likely are expressed at low levels in the cells. This was further demonstrated in 5′ RACE experiments using Marathon-Ready cDNA from brain (Clontech) in which only 2 of 30 transcripts sequenced contained exon 3 (data not shown). Using Real-Time qPCR technique we sought to quantify the amount of exon 3 containing transcripts relative to the total level of *ENDOV* transcripts in brain tissue and primary fibroblasts. For this purpose we designed primers specific for exon 3 (Table S1: primers 1 and 2) and exon 7 (representing the total amount of transcript, Table S1: primers 3 and 4), and standard curve measurements confirmed an equal amplification efficiency of ∼95% for the two primer sets. It was shown that in cDNA from brain tissue and HE fibroblasts the amount of transcripts containing exon 3 was approximately 30% and 50% relative to exon 7 containing transcripts, respectively. These data support the findings from the sequence databases analyses showing that human *ENDOV* transcripts without exon 3 are more abundant than the transcripts that contain exon 3.

### *ENDOV* expression during cell cycle

**Figure 2:**
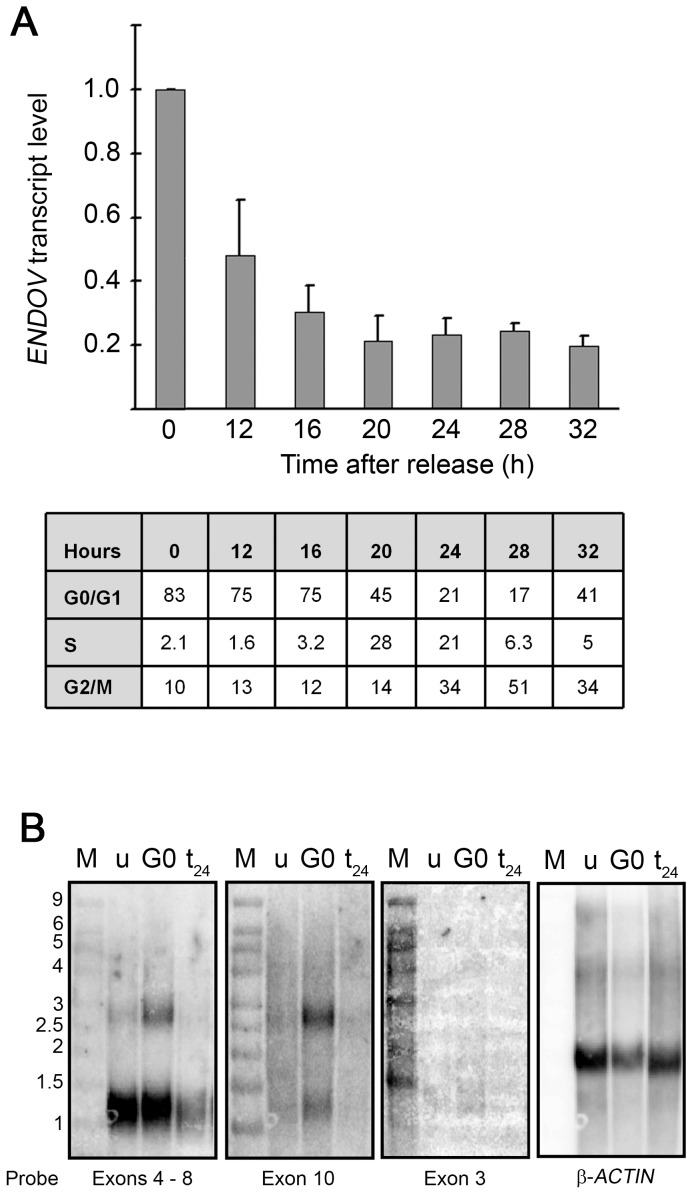
Upregulation of *ENDOV* transcription during quiescence. (**A**) Human embryonic fibroblasts were arrested in G0 by serum starvation at confluence and released by replating 1∶4 in culture medium with serum. *ENDOV* transcript levels (exons 2 to 3) were measured during cell cycle progression after G0 release at indicated time points by qRT-PCR. G0 cells were used as the reference for calculations. The average of 3 parallels (same RNA) was calculated and standard deviation is shown. Cell cycle distribution was monitored using propidium iodide staining followed by flow cytometry after release from the block. The percentage of cells in each cell cycle is presented in the table. The experiment was repeated twice with similar results. (**B**) Nothern blot analysis of *ENDOV* mRNA. mRNA was isolated from human fibroblasts that were unsynchronised (u), G0 arrested (G0) and allowed to proliferate for 24 hours (t_24_), separated by electrophoresis and transferred to a nylon membrane. Hybridisation signals with probes spanning exons 4–8, exon 10, exon 3 of *ENDOV* and for β*-ACTIN* are shown. M is the RNA size standard as indicated (in kilobases).

To examine whether *ENDOV* transcription is regulated during cell-cycle progression, cultured human fibroblasts were arrested in G0 by contact inhibition and serum deprivation. Flow cytometry showed that 83% of the cells were in G0/G1 phase (Figure 2A). Cells were released from the arrest by culturing at subconfluence in serum supplemented complete medium and harvested at time points as indicated. Total RNA was isolated and Real-Time qRT-PCR experiments were performed with exon 3 specific primers (Table S1: primers 1 and 2). The results revealed highest level of *ENDOV* mRNA in G0 arrested cells and a reduction upon cell cycle progression (Figure 2A). After 20–28 h, where most of the cells were in S or G2/M phases, the *ENDOV* transcript level had decreased 4–5 folds.

To further investigate *ENDOV* transcripts, northern blot analysis was performed with mRNA isolated from HE fibroblasts synchronised as above. A probe spanning exons 4–8 gave a strong signal corresponding to transcripts of 1200–1400 nucleotides (nt) in addition to a weaker band of 2800 nt (Figure 2B). A transcript of 2800 nt corresponds to the full-length transcript (1–10) which was verified with an exon 10 specific probe (Figure 2B). This signal was highest in G0 arrested cells (3–4 fold), confirming the qRT-PCR data. However, the majority of the transcripts (∼70%) detected with the exon 4–8 probe, was less than 1500 nt and appeared to lack exon 10. With an exon 3 specific probe, two faint bands of ∼1200 and ∼1400 nt were detected, but none of 2800 nt (Figure 2B). As the signals were very weak, it might be that the amount of full-length transcripts is below the detection limit with this specific probe. Thus, our expression analyses show that the *ENDOV* mRNA level is steady in cycling cells but peaks upon quiescence. Further, different *ENDOV* transcript variants exist and the majority appears to lack exon 10.

In order to clarify which protein variants are produced by the cells, we attempted to immunoprecipitate endogenous ENDOV by the use of an in-house rabbit polyclonal antibody raised against ENDOV mixed with protein extract made from G0 arrested HE fibroblasts. However, we were not able to precipitate ENDOV as evidenced by lack of signals in western blot analysis (data not shown). It appears that the expression of ENDOV is below the detection limit of this method.

### *ENDOV* expression in various tumor samples

**Figure 3:**
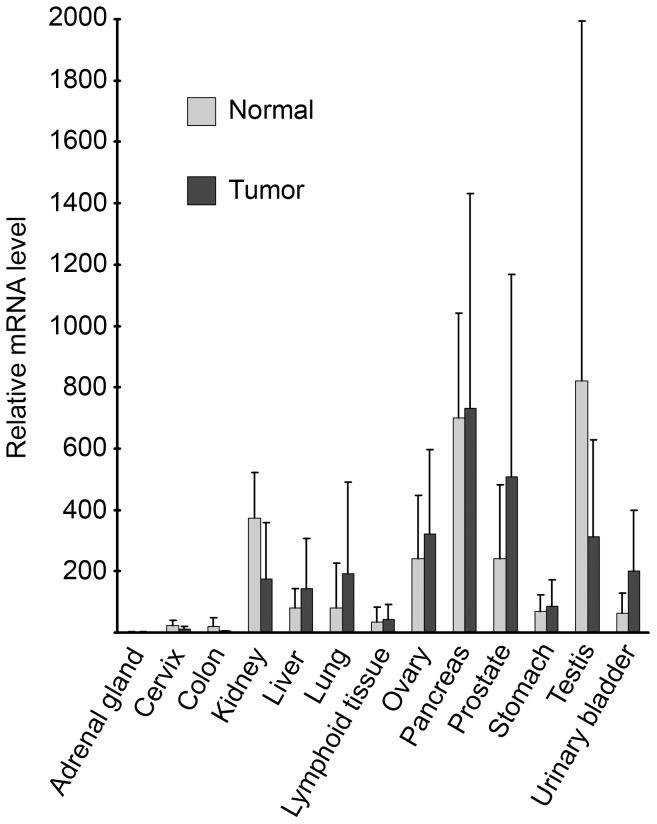
Expression of *ENDOV* in human cancers. Transcript profiling was performed using a cancer tissue qPCR array and primers amplifying exons 6 to 8 of *ENDOV*. cDNA levels are normalised to β*-ACTIN*. *ENDOV* mRNA levels were calculated relative to the tissue with lowest expression level (adrenal gland). Tumour and normal tissue were grouped according to their origin and the average and standard deviation were calculated for each group. The experiment was performed twice with similar results.

We also investigated the expression level of *ENDOV* in several tumor types and their normal counterparts by using qPCR arrays containing cDNA from diseased and normal tissues and primers for exons 6/7 to 8. Although we observed high variation within the samples from the same tissue, the results showed that tumor samples in general did not display altered expression of *ENDOV* compared to the corresponding normal tissues (Figure 3). In addition, this analysis showed that kidney, pancreas and testis tissue express highest amounts of *ENDOV* whereas adrenal gland, cervix and colon are the tissues with lowest level of *ENDOV*.

### ENDOV localizes to nucleolus

**Figure 4:**
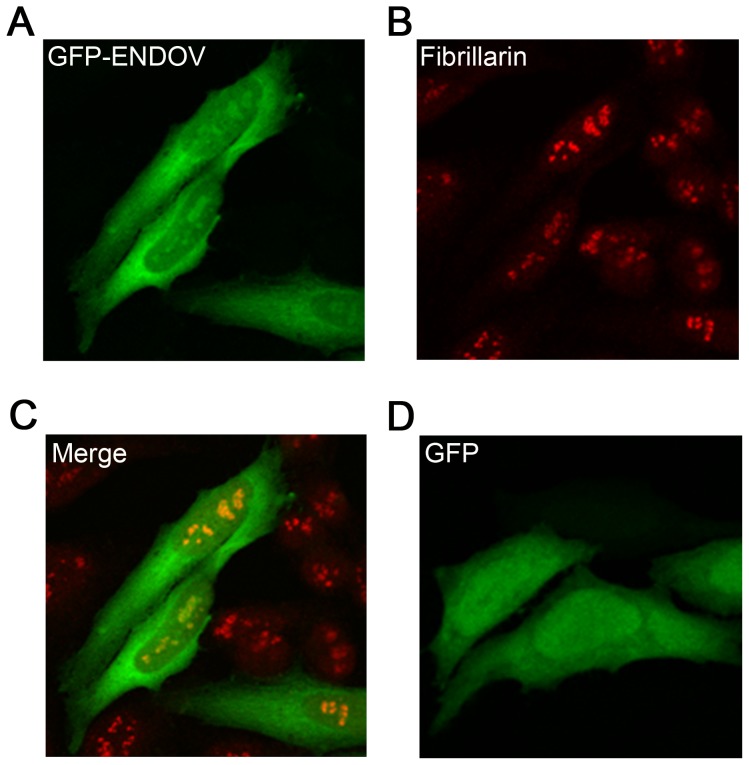
The EGFP-ENDOV fusion protein localises to nucleoli and cytoplasm. Confocal imaging of HeLa cells overexpressing GFP-ENDOV (**A**) probed with an anti-fibrillarin antibody (**B**), an overlay of the two (**C**) or GFP only (**D**).

Cellular localization of ENDOV was examined in HeLa cells transiently transfected with full-length *ENDOV* (1–10) cloned into pEGFPN/C vectors. The GFP-ENDOV fusion protein was found in the cytoplasm and in nucleolus, confirmed by colocalization with the nucleolar protein fibrillarin (Figure 4 A–C). The same localization pattern was also seen when the GFP-tag was placed at the C-terminal end of the protein (data not shown). The GFP protein itself was evenly distributed throughout the cell (Figure 4D). Western blot analysis of protein extracts from the GFP-ENDOV expressing cells probed with a GFP antibody, showed that GFP-ENDOV existed as fusion proteins migrating slightly faster than the expected sizes (MW GFP: 27 kD, ENDOV: 30.8 kD) (Figure S2). These data demonstrate that full-length ENDOV may exert its function in nucleolus and possibly also in cytoplasm.

### ENDOV has no endonuclease activity but binds branched DNA structures

The primary activity of prokaryotic endoV enzymes is incision of the second phosphodiester bond 3′ to Hx residues in the DNA. To test whether this also is true for the human homolog, we performed endonuclease activity assays with recombinant ENDOV (1–10) and an Hx containing DNA substrate. Unexpectedly, ENDOV had no activity towards Hx under the same condition where *E. coli* endoV cleaved efficiently (Figure S3). Different reaction conditions were tested by varying pH (6.5–8.5) and salt ions (Mg^2+^, Mn^2+^) without any effect on ENDOV endonuclease activity. *E. coli* endoV is also active on uracil [17] and AP site [14] containing DNA, however no cleavage was obtained when ENDOV was tested against these substrates (data not shown).

**Figure 5:**
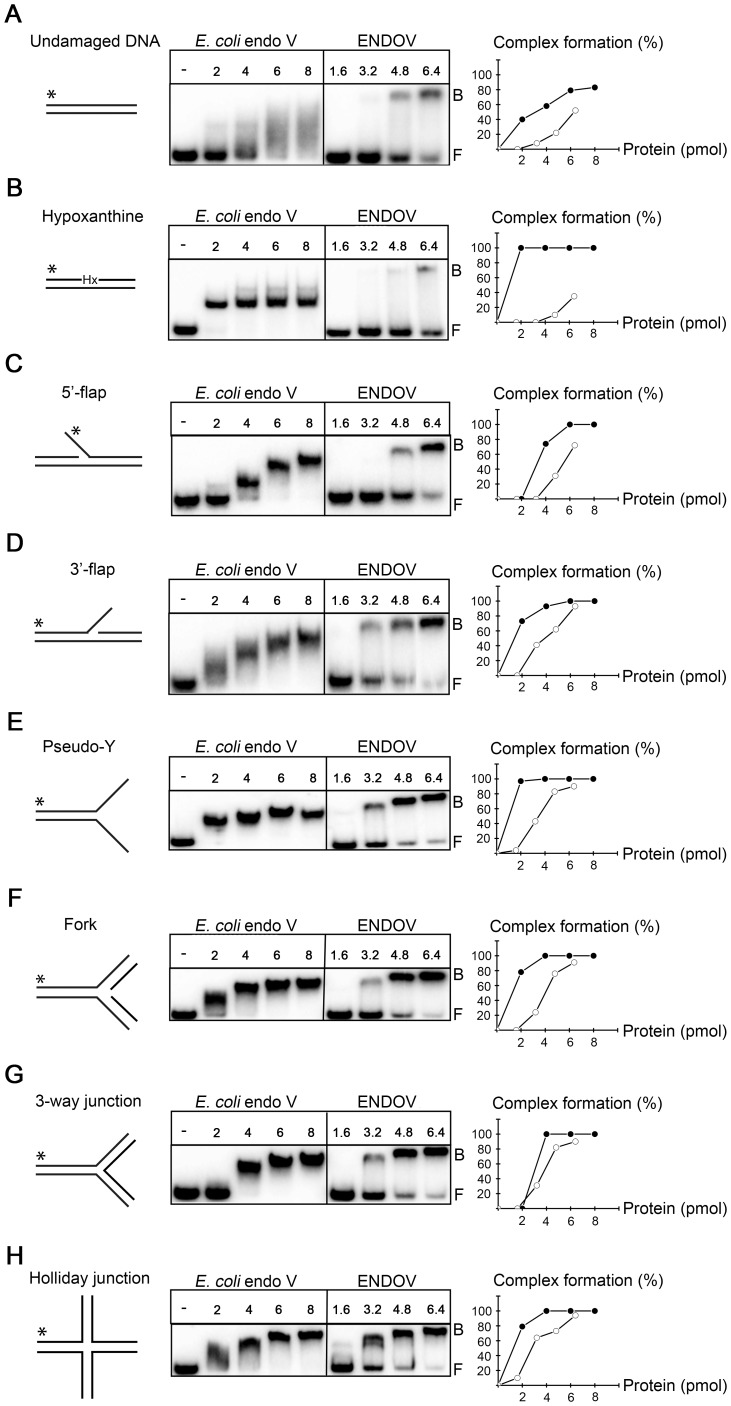
Human ENDOV binds branched DNA substrates. The affinities of *E. coli* and human endonuclease V for different DNA substrates were tested by electrophoretic mobility shift assay. Substrates used were: (**A**): Undamaged DNA, (**B**): Hypoxanthine, (**C**): 5′-flap, (**D)**: 3′-flap, (**E**): pseudo-Y, (**F**): fork, (**G**): 3-way junction and (**H**): Holliday junction (asterisk indicates the ^32^P-labelled strands). 2–8 pmol *E. coli* endoV and 1.6–6.4 pmol ENDOV enzymes were assayed with 10 fmol substrates as indicated. All experiments were repeated 2 to 3 times and a representative assay is shown. Bound substrates relative to free were quantified and are shown to the right. F = free DNA, B = bound DNA, - = no enzyme added, filled circles = *E. coli* endoV; open circles = ENDOV.

**Figure 6:**
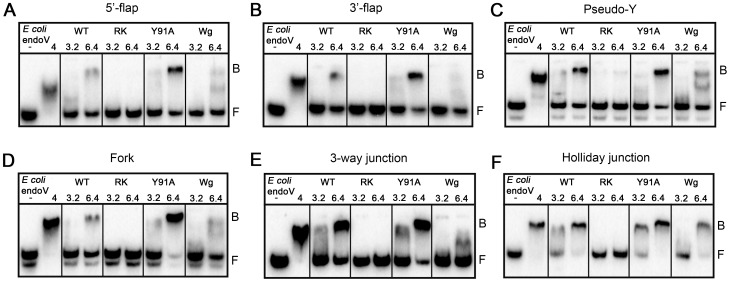
The RK ENDOV mutant has lost its affinity for branched DNA substrates. Three ENDOV site specific mutants, RK (R248E/K249E double mutant), Y91A, and Wg (residues P90–S93 replaced with 4 glycins), wild type enzyme (3.2 and 6.4 pmol) and *E. coli* endoV (4 pmol) were tested for their ability to bind branched DNA substrates. Substrates tested were: (**A**): 5′-flap, (**B**): 3′-flap, (**C**): pseudo-Y, (**D**), fork, (**E**): 3-way junction and (**F**) Holliday junction. F = free DNA, B = bound DNA, - = no enzyme added.

**Figure 7:**
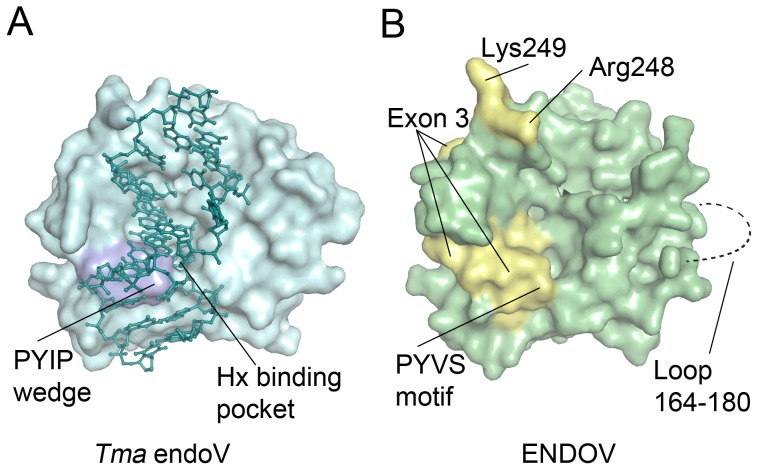
Location of structural elements in endonuclease V. (**A**) Structure of *Tma* endoV binding to deaminated DNA (PDB code 2W35 [23]) showing the central location of the strand-separating wedge (PYIP motif) close to the damage recognition pocket. (**B**). Homology model of human ENDOV showing the location of mutated residues Arg248 and Lys249 (yellow), as well as residues forming the PYVS motif in exon 3 (yellow), which were also mutated. A loop comprising residues 164–180 could not be reliably modelled and is not included (dashed line).

In addition to deaminated bases, *E. coli* endoV is also active upon different branched DNA structures such as 5′-flaps, hairpins and insertion/deletions loops where it cleaves 3′ to the branching point [21]. Human ENDOV was tested against a panel of similar DNA substrates including 5′-flap, 3′-flap, 3-way junction, pseudo-Y, fork and Holliday junction, but no endonuclease activity was detected on these substrates either (data not shown). Next, we tested whether ENDOV could recognize and bind any of these substrates. Only very weak and unspecific binding was found for the Hx containing DNA and for the undamaged double stranded DNA substrate (Figure 5A, B). In contrast, ENDOV had high affinity for all 6 branched DNA substrates (Figure 5C–H). The affinities of *E. coli* endoV for these substrates were comparable to Hx containing DNA. To our knowledge, binding of *E. coli* endoV to 3′-flap, fork, 3-way and Holliday junction has not previously been demonstrated. For *E. coli* endoV with control double stranded DNA, a smear rather than a distinct shift was observed, possibly reflecting weak interactions between the DNA and enzyme that cease during gel electrophoresis. To biochemically map the binding between ENDOV and branched DNA, three different site-specific mutants were designed and the corresponding proteins purified after heterologous expression in *E. coli*. All three mutant proteins were soluble and expressed to the same level as the wild type protein (Figure S4). The Tyr73/80 in *E. coli* and *T. maritima* endoV enzymes are key residues for Hx strand recognition [39], however substitution of the corresponding ENDOV Y91 to an alanine had no effect on the binding affinity to branched substrates (Figure 6A–F), suggesting that the Tyr residue of the wedge structure is not involved in recognition of branched DNA structures. However, the wedge mutant ENDOV-Wg, with the entire DNA strand-separating motif PYVS (residue 90–93) replaced with 4 glycin residues, was strongly compromised in DNA binding. It appears that removal of the entire wedge domain introduces major structural distortions that abolish accommodation of DNA. Further, the ENDOV double mutant RK, with amino acids Arg248 and Lys249, predicted to be involved in sugar-phosphate binding based on comparison with the structure of *T. maritima* endoV (Figure 7), replaced by glutamate residues, completely lost the DNA binding ability. In sum, these data suggest that human ENDOV employ the same mechanism as its prokaryotic counterparts to accommodate branched DNA, whereas the ability to recognize Hx is severely diminished.

## Discussion

Deamination of adenine to Hx in DNA occurs endogenously at a low but significant level and may result in A∶T to G∶C transition mutations. Removal of Hx is therefore necessary for the maintenance of genomic integrity and the primary enzyme involved in this process in *E. coli* is endoV. Homologs of endoV are present in all kingdoms of life and represent a family of highly conserved proteins (Figure S1). The high degree of evolutionary conservation clearly demonstrates that these proteins have an important functional role in the cells. Nevertheless, the functions of the eukaryotic endoV homologs remain unknown. Here we have characterized human ENDOV by gene expression, subcellular localization and biochemical analysis.

Information available in public databases shows that human *ENDOV* transcripts are highly variable. Surprisingly, most of the transcripts, experimentally determined by many different groups, are highly unlikely to encode functional protein. Our gene expression experiments confirmed the existence of multiform, truncated and probably misspliced transcripts in human cells. The lack of exon 3 in many transcripts could be due to an unfavorable splice donor site in intron 3 (GT substituted with GC) which may allow splicing of exon 4 directly to exon 2. The nonstandard GC-AG intron 3 is conserved in eutherian mammals, except rodents, but not in marsupials and other vertebrates. Whether this nonstandard intron contributes to *ENDOV* transcript regulation in eutherian cells is at present unknown. By RT-PCR, we demonstrated that transcripts containing the expected exon 2-exon 3 junction are present in human cells, although at significantly lower level than total *ENDOV* mRNA. Nevertheless, at least some full-length transcripts appear to be present in most cell types. Northern blot analysis showed that the majority of transcripts were 1200–1400 nt long. This probably corresponds to an *ENDOV* transcript containing exon 9 and lacking exon 10, with identical splicing as for example in mouse, pig, and sea urchin (Figure 1A). Less abundant is a larger transcript hybridizing to an exon 10 specific probe which could correspond to an isoform harboring a short exon 9 in addition to exon 10 (2800 nt). Variation in the 3′ end of the *ENDOV* transcript allows for different C-termini of the expressed protein. Whereas amino acids 1–250, encoded by exons 1–8, constitute the structurally ordered core domain required for ENDOV function in prokaryotes, the C-terminus is structurally flexible and apparently evolving neutrally. Prokaryotic proteins, as well as the orthologs from frog, *S. pombe* and several other eukaryotes (Figure S1) do not possess the extended C-terminus, suggesting that it may have no significant function. Both the biochemical analyses and localization studies were performed with the two ENDOV isoforms 1–9 (data not shown) and 1–10, always with similar results, supporting a non-essential role for the C-terminus.

Prokaryotic endonuclease V proteins are considered the principal enzyme for Hx repair and quite surprisingly we were unable to demonstrate such an activity for human ENDOV. It was previously shown that mouse ENDOV processes Hx in single stranded DNA. However, to get cleavage of a double stranded Hx substrate, micromolar concentrations of the enzyme was needed [28], questioning the efficiency of the Hx activity also for mouse ENDOV. *E. coli* endoV and the other characterized bacterial endoV enzymes have clearly a high and robust Hx nicking activity *in vitro*, but their role *in vivo* is more unclear. For instance, *E. coli* cells lacking endoV have no pronounced phenotype except an increased mutation frequency when exposed to nitrous acid [16]. In *E. coli*, another enzyme, the AlkA DNA glycosylase, also acts on Hx in DNA [40], suggesting redundancy in the repair of Hx. Conversely, deletion of *alkA* in an *nfi* mutant does not increase the mutation frequency of the *nfi* cells questioning the assumption of redundancy [16]. Another distinct role for *E. coli* endoV was found by analysis of the deoxyinosine triphosphatase *rdgB* mutant, which in combination with a *recA* mutant is lethal. RgdB functions in degradation of dITP and hence, an *rdgB* mutant has increased levels of dITP in the nucleotide pool and consequently also Hx in the DNA. The lethality of the double mutant is believed to be associated with endoV induced strand breaks at Hx which cannot be repaired in the recombination defect *recA* background. Simultaneous deletion of *nfi* suppresses the lethality, pointing to a clear role for endoV under these circumstances. It appears that DNA strand incision is important, but none of these studies demonstrate that Hx actually is removed from DNA. In fact, a recent study shows that the genes affecting the Hx levels in DNA all belong to the purine nucleotide metabolism whereas *nfi* has no effect [41].

In this work we were not able to demonstrate endonuclease activity for recombinant human ENDOV. We cannot exclude that this is due to a missing factor or protein partner interacting with ENDOV. The dependence on associated proteins for activity has been shown for several endonucleases such as MUS81, SLX1 and XPF interacting with EMM1 [42]–[44], SLX4 [44]–[46] and ERCC1 [47], respectively. Interestingly, these are all structure-specific endonucleases active upon the types of DNA substrates used in this work. We may speculate if the sorting to the nucleoli and the affinity for specific DNA structures such as flaps and branching points could hint to a role of ENDOV in replication or transcription of ribosomal DNA. The rDNA genes are organized in long tandem repeats in hundreds of copies that are dynamic in size (reviewed in [48]). Tight control of replication is required to avoid unwanted expansions or contractions. Moreover, replication fork barriers are present in nontranscribed regions which prevent collisions between replication forks and the transcription machinery (reviewed in [49]). Both these processes have the potential to create DNA structures that are possible substrates for ENDOV.

*T. maritima* endoV has much stronger affinity for Hx-DNA than non-damaged DNA and the protein crystal structure shows that the conserved PYIP wedge is important for presenting the Hx base into the lesion recognition pocket [23]. It appears that human ENDOV have lost the ability to efficiently recognize Hx in DNA although the wedge structure and the base recognition pocket appear to be conserved. However, the binding to branched DNA remains intact in human ENDOV, suggesting that the structural fold may have been evolutionary modified to avoid incision at Hx in DNA in mammalian cells.

During preparation of this report, Mi *et al.* [50] published a paper describing biochemical properties of recombinant ENDOV. In contrast to our data, they find Hx nicking activity, albeit weak, for human ENDOV. We do not know the reason for this discrepancy, but the use of a fusion protein of ENDOV with thioredoxin in their study may influence the results.

## Supporting Information

Figure S1 **Multiple sequence alignment of 14 eukaryotic endonuclease V homologs.** The sequences are from human (RefSeq [36] identifier NP_775898), mouse (*Mus musculus*, NP_001158108), hamster (*Cricetulus griseus*, XP_003496919), rat (*Rattus norvegicus*, GenBank [29] identifier EDM06795), pig (*Sus scrofa*, XP_003131183), chicken (*Gallus gallus*, XP_420082), frog (*Xenopus tropicalis*), medaka ricefish (*Oryzias latipes*), sea urchin (*Strongylocentrotus purpuratus*, XP_794487), hemichordate acorn worm (*Saccoglossus kowalevskii*, XP_002731652), sponge (*Amphimedon queenslandica*, XP_003386872), *Trichoplax adhaerens* (XP_002110086), the fission yeast *Schizosaccharomyces pombe* (NP_594332), and *Arabidopsis thaliana* (NP_567868). In addition, the sequences of the bacterial homologs from *E. coli* (NP_418426) and *Thermotoga maritima* (NP_229661) are shown. The medaka sequence was generated from Ensembl [31] protein ENSORLP00000002194 (exons 1–7), but with the C-terminus derived from EST sequences [29] BJ009743 and BJ023154. The full-length *X. tropicalis* sequence was generated by combining data from IMAGE cDNA clone sequences BC154886 and BC087745 and several ESTs (*e.g.* DN064695). The alignment was generated with Muscle [34]. Conserved and functionally important residues are highlighted above and below the alignment for the human (black) and *T. maritima* (dark blue) endoV homologs, respectively. These includes the residues of the DDD-motif of the catalytic triad (human residues Asp52, Asp126, and Asp240) which together with Glu100 are complexing the divalent cation of the catalytic site, the residues forming the lesion recognition pocket (Tyr91, Gly94, Leu96, Gly127, Asn128, His132, Gly137, and Leu158), as well as the active site stabilizing Lys155. See Dalhus *et al.* [23] for more details on lesion recognition and the catalytic mechanism of endoV.
(JPG)

Figure S2 **GFP-ENDOV exists as a fusion protein.** Protein extracts were prepared from HeLa cells overexpressing (from left) GFP alone or GFP-ENDOV. Proteins were separated on 10% SDS-PAGE in 1× MOPS and transferred to PVDF membranes as described in Material and methods. The membrane was probed with a GFP antibody. M is the molecular weight marker as indicated.
(JPG)

Figure S3 **Human ENDOV does not cleave a hypoxanthine containing DNA substrate.** *E. coli* endoV (2–8 pmol) and ENDOV (1.6–6.4 pmol) were tested for activity towards hypoxanthine DNA. Reaction products were separated by PAGE and visualised by Phosphorimaging. S = substrate, C = cleaved substrate, - = no enzyme added.
(JPG)

Figure S4 **SDS-PAGE analysis of purified** ***E. coli*** **endoV, human ENDOV wildtype (WT) and mutant (RK, Y91A, Wg) proteins.** 1 µg of each protein was analysed by SDS-PAGE. M is the molecular weight marker as indicated.
(JPG)

Table S1 **Oligonucleotides for probes and real time-PCR.**
(PDF)

Table S2 **Oligonucleotides for DNA substrates.**
(PDF)

Table S3 **Evaluation of the evolutionary selective pressure acting on ENDOV.**
(PDF)
