# Supplementary figures and images for: The Human Homolog of Escherichia coli Endonuclease V Is a Nucleolar Protein with Affinity for Branched DNA Structures
Source: PLoS One. 2012 Nov 5;7(11):e47466. doi: 10.1371/journal.pone.0047466 (PMC3489907; doi:10.1371/journal.pone.0047466)

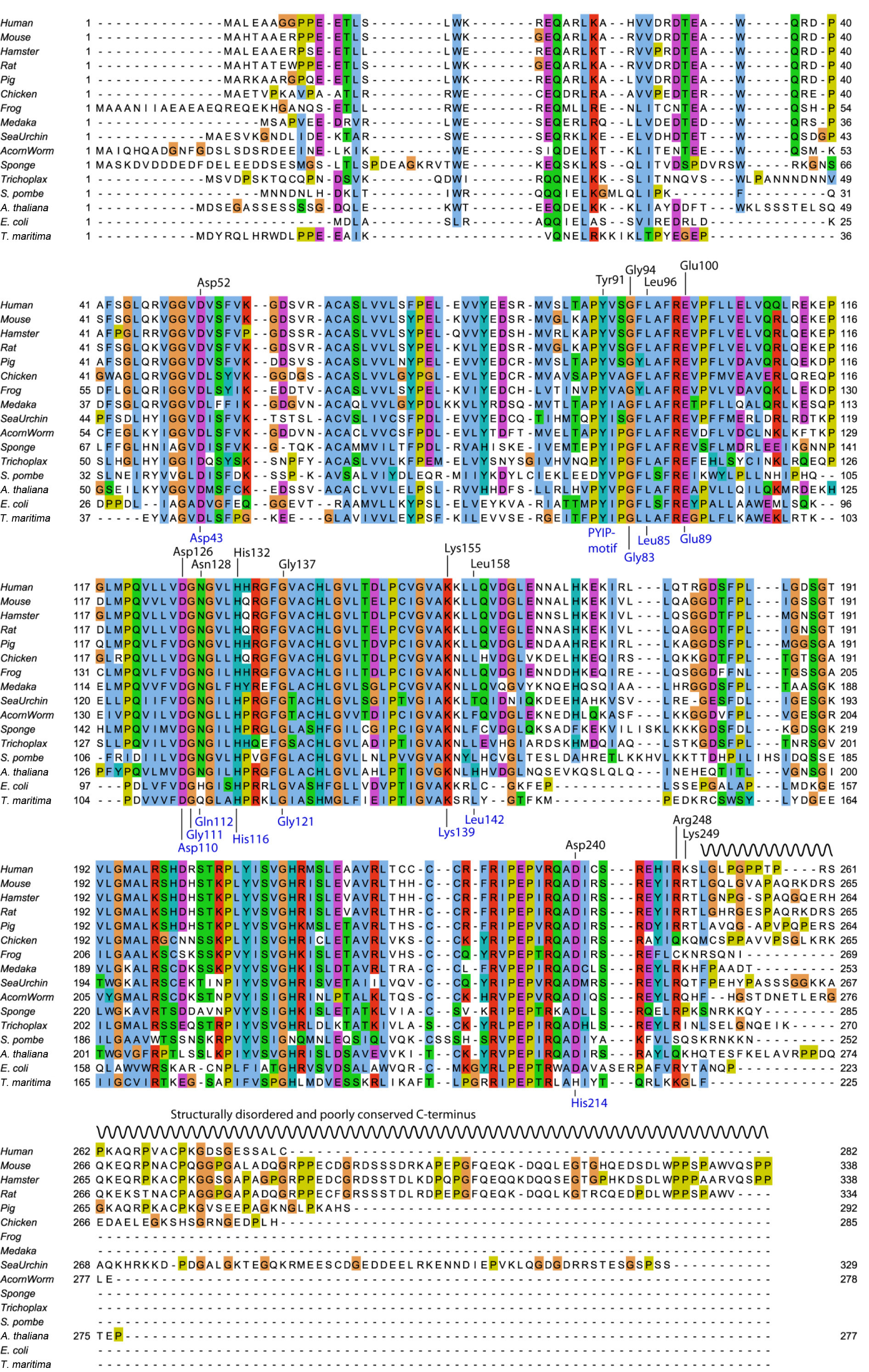

Supplement: Figure S1 — Multiple sequence alignment of 14 eukaryotic endonuclease V homologs. The sequences are from human (RefSeq [36] identifier NP_775898), mouse (Mus musculus, NP_001158108), hamster (Cricetulus griseus, XP_003496919), rat (Rattus norvegicus, GenBank [29] identifier EDM06795), pig (Sus scrofa, XP_003131183), chicken (Gallus gallus, XP_420082), frog (Xenopus tropicalis), medaka ricefish (Oryzias latipes), sea urchin (Strongylocentrotus purpuratus, XP_794487), hemichordate acorn worm (Saccoglossus kowalevskii, XP_002731652), sponge (Amphimedon queenslandica, XP_003386872), Trichoplax adhaerens (XP_002110086), the fission yeast Schizosaccharomyces pombe (NP_594332), and Arabidopsis thaliana (NP_567868). In addition, the sequences of the bacterial homologs from E. coli (NP_418426) and Thermotoga maritima (NP_229661) are shown. The medaka sequence was generated from Ensembl [31] protein ENSORLP00000002194 (exons 1–7), but with the C-terminus derived from EST sequences [29] BJ009743 and BJ023154. The full-length X. tropicalis sequence was generated by combining data from IMAGE cDNA clone sequences BC154886 and BC087745 and several ESTs (e.g. DN064695). The alignment was generated with Muscle [34]. Conserved and functionally important residues are highlighted above and below the alignment for the human (black) and T. maritima (dark blue) endoV homologs, respectively. These includes the residues of the DDD-motif of the catalytic triad (human residues Asp52, Asp126, and Asp240) which together with Glu100 are complexing the divalent cation of the catalytic site, the residues forming the lesion recognition pocket (Tyr91, Gly94, Leu96, Gly127, Asn128, His132, Gly137, and Leu158), as well as the active site stabilizing Lys155. See Dalhus et al. [23] for more details on lesion recognition and the catalytic mechanism of endoV. (JPG) [file pone.0047466.s001.jpg]

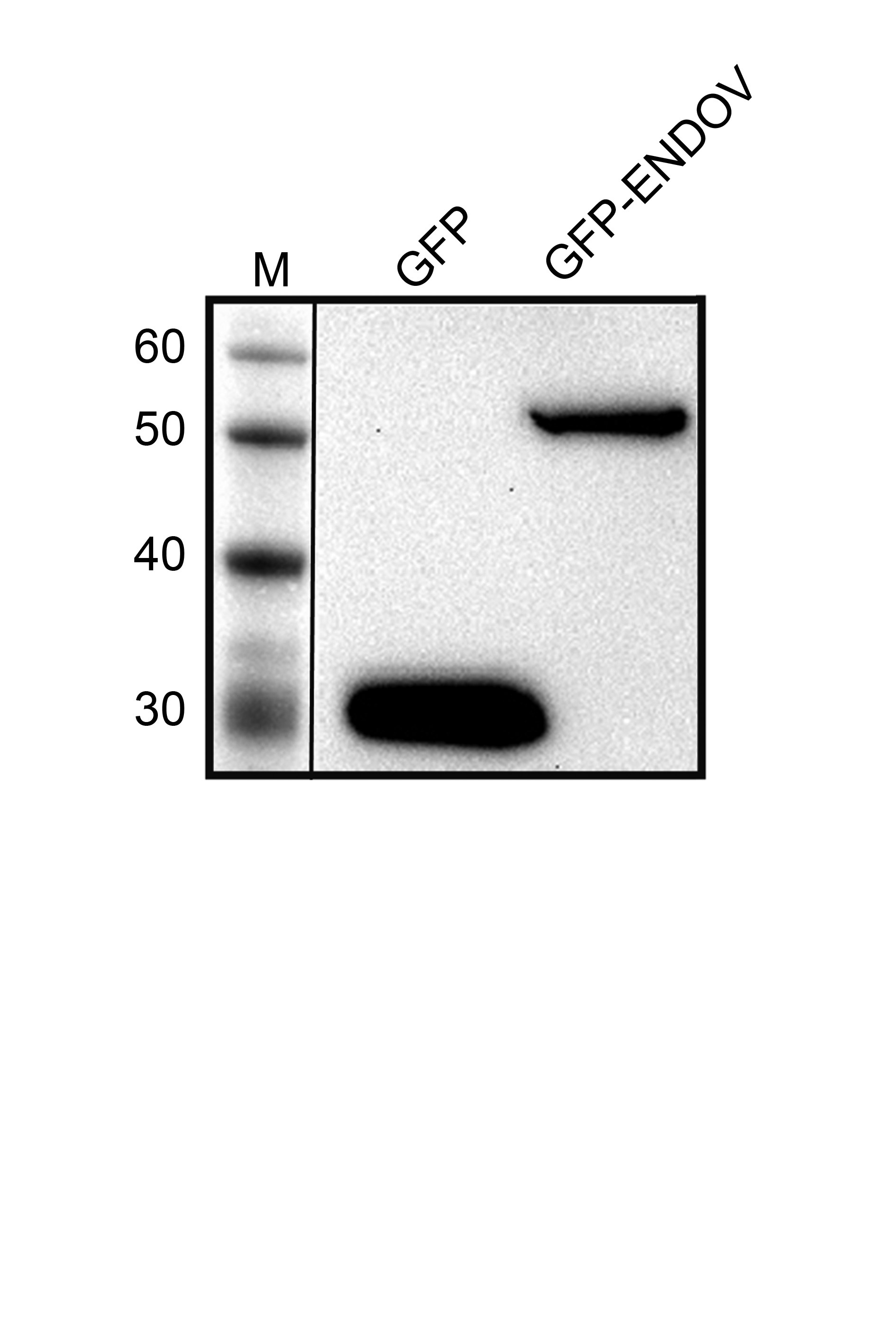

Supplement: Figure S2 — GFP-ENDOV exists as a fusion protein. Protein extracts were prepared from HeLa cells overexpressing (from left) GFP alone or GFP-ENDOV. Proteins were separated on 10% SDS-PAGE in 1× MOPS and transferred to PVDF membranes as described in Material and methods. The membrane was probed with a GFP antibody. M is the molecular weight marker as indicated. (JPG) [file pone.0047466.s002.jpg]

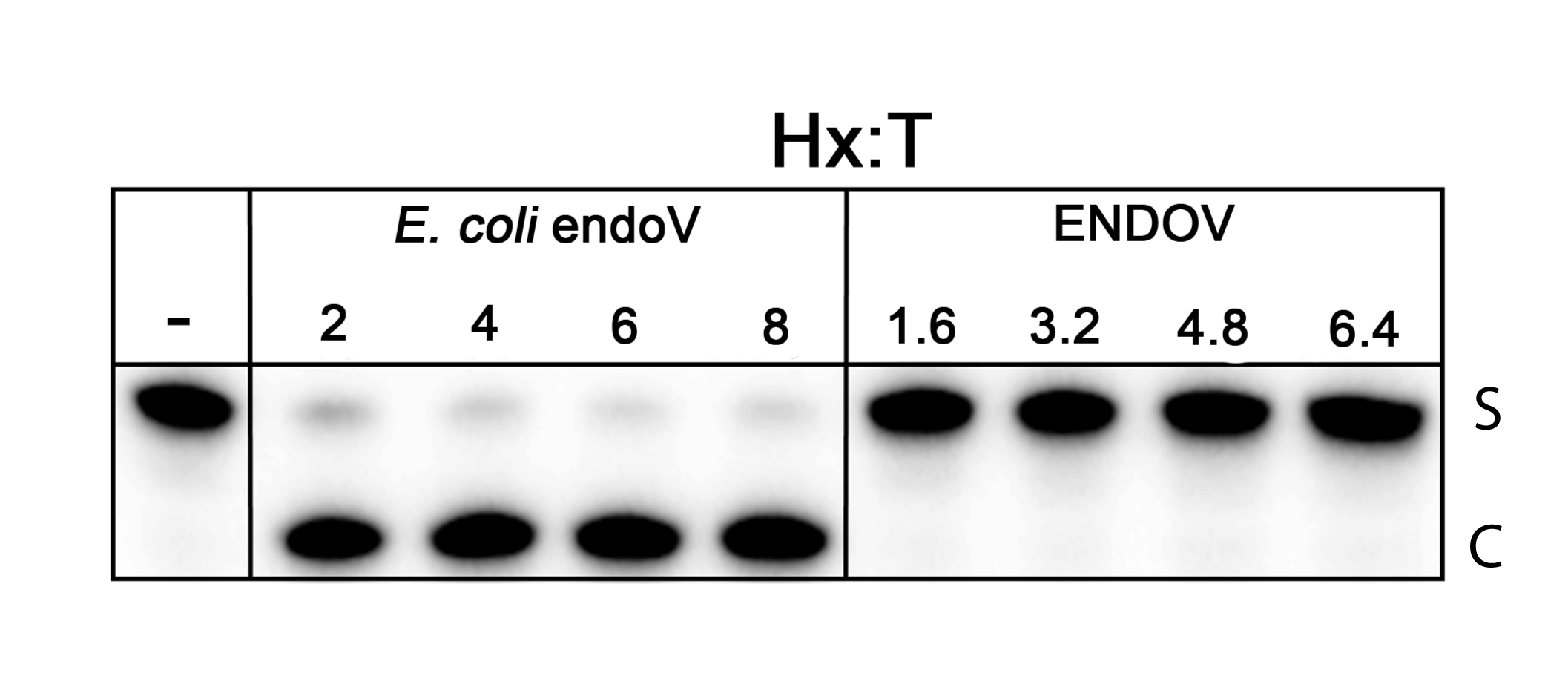

Supplement: Figure S3 — Human ENDOV does not cleave a hypoxanthine containing DNA substrate. E. coli endoV (2–8 pmol) and ENDOV (1.6–6.4 pmol) were tested for activity towards hypoxanthine DNA. Reaction products were separated by PAGE and visualised by Phosphorimaging. S = substrate, C = cleaved substrate, - = no enzyme added. (JPG) [file pone.0047466.s003.jpg]

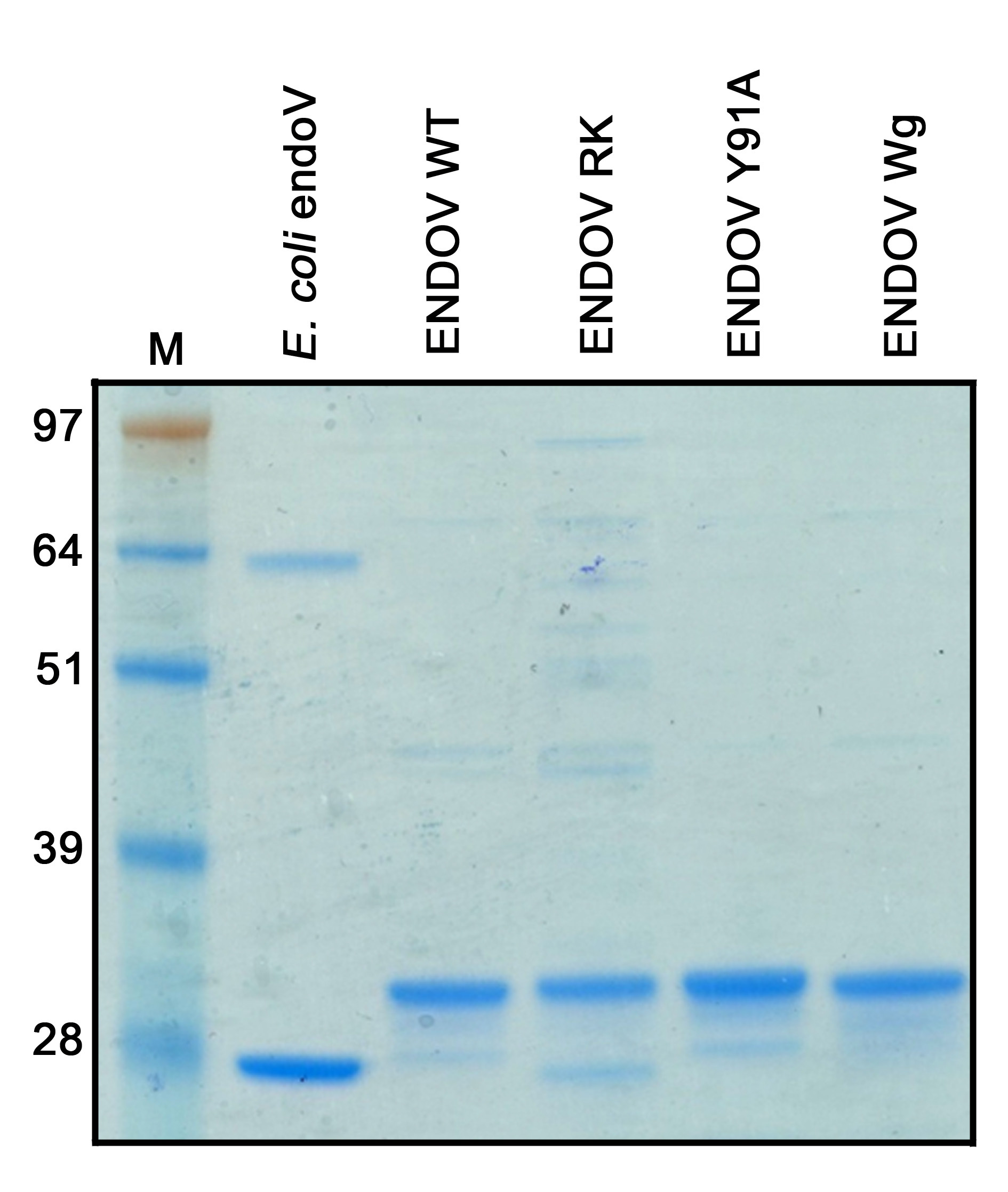

Supplement: Figure S4 — SDS-PAGE analysis of purified E. coli endoV, human ENDOV wildtype (WT) and mutant (RK, Y91A, Wg) proteins. 1 µg of each protein was analysed by SDS-PAGE. M is the molecular weight marker as indicated. (JPG) [file pone.0047466.s004.jpg]
