# Supplementary material for: The Human Homolog of Escherichia coli Endonuclease V Is a Nucleolar Protein with Affinity for Branched DNA Structures
Source: PLoS One. 2012 Nov 5;7(11):e47466. doi: 10.1371/journal.pone.0047466 (PMC3489907; doi:10.1371/journal.pone.0047466)
Supplement: Table S1 — Oligonucleotides for probes and real time-PCR. (PDF) [file pone.0047466.s005.pdf]

Table S1. Oligonucleotides for probes and real time-PCR

Primer:	Sequence 5' → 3':	Description:
1	TTGACGTGTCCTTCGTGAAA	Fwd exon 2 RT-PCR
2	ATGCGGCTCTCCTCATACAC	Rev exon 3 RT-PCR
3	ACTGTCCTGGGAATGGC	Fwd exon 6/7 RT-PCR, tissue-scan
4	ACTTGCGGATGTGCTCTCG	Rev exon 8 RT-PCR, tissue-scan
5	CCACATCGCTCAGACACCAT	Fwd GAPDH RT-PCR
6	GCGCCCAATACGACCAAAT	Rev GAPDH RT-PCR
7	GTGGTGTATGAGGAGAGCCG	Fwd exon 3 Northern
8	CTGGGGCATGAGGCCCG	Rev exon3 Northern
9	CCTTCTTGTGGATGGAAACGGGGTAC	Fwd exon 4 Northern
10	AGCGACTTGCGGATGTGCTCTCG	Rev exon 8 Northern
11	TGGTGGTGAGAGCACACGTC	Fwd exon 10 Northern
12	GGACCGTCTTCAGCTGGATG	Rev exon 10 Northern
