# Supplementary material for: The Human Homolog of Escherichia coli Endonuclease V Is a Nucleolar Protein with Affinity for Branched DNA Structures
Source: PLoS One. 2012 Nov 5;7(11):e47466. doi: 10.1371/journal.pone.0047466 (PMC3489907; doi:10.1371/journal.pone.0047466)
Supplement: Table S2 — Oligonucleotides for DNA substrates. (PDF) [file pone.0047466.s006.pdf]

# Table S2. Oligonucleotides for DNA substrates

| ***Primer:*** | ***Sequence 5' → 3':*** | ***Description:*** |
|---|---|---|
| 1 | CGGTGACCGATCTGTAGCTCTACGG | Complementary oligo for ds control and Hx |
| 2 | CCGTAGAGCTACAGATCGGTCACCG | ds control* |
| 3 | CCGTAGAGCTAC[Hx]GATCGGTCACCG | Hypoxanthine* |
| 4 | GCATGCCTGCACGG[U]CATGGCCAGATCCCCGGGTACCGAG | Uracil* |
| 5 | CTCGGTACCCGGGGATCTGGCCATGGCCGTGCAGGCATGC | Complementary G for uracil |
| 6 | CCGTAGAGCGACAGATCGGTCACCG | Complementary for loop (A)* |
| 7 | CGGTGACCGATCTTGTCGCTCTACGG | Loop (TT) |
| 8 | TCAACTCTGGAATAAGTGCGTGGTCGGT | Complementary oligo for hairpin* |
| 9 | ACCGACCACGCACTGCGCGTTTTCGCGCTATTCCAGAGTTGA | Hairpin |
| 10 | GGATACGTAACAACGCTTATGCATCGCCGCCGCTACATCCCTGAGCTGAC | 3'-flap, 5'-flap, 3-way*, pseudo-Y*, fork |
| 11 | ATGCATAAGCGTTGTTACGTATCC | 3'-flap, fork |
| 12 | GTCAGCTCAGGGATGTAGCGGCGG | 5'-flap |
| 13 | TGTGTTCGATCTCGATCAGAATGACGATGCATAAGCGTTGTTACGTATCC | 5'-flap*, 3-way, pseudo-Y |
| 14 | GTCAGCTCAGGGATGTAGCGGCGGAGTCATTCTGATCGAGATCGAACACA | 3'-flap*, 3-way, fork* |
| 15 | TGTGTTCGATCTCGATCAGAATGA | fork |
| 16 | CCGCTACCAGTGATCACCAATGGATTGCTAGGACATCTTTGCCCACCTGCAGGTTCACCC | HJ_1 |
| 17 | TGGGTGAACCTGCAGGTGGGCAAAGATGTCCTAGCAATCCATTGTCTATGACGTCAAGCT | HJ_2* |
| 18 | GAGCTTGACGTCATAGACAATGGATTGCTAGGACATCTTTGCCGTCTTGTCAATATCGGC | HJ_3 |
| 19 | TGCCGATATTGACAAGACGGCAAAGATGTCCTAGCAATCCATTGGTGATCACTGGTAGCGG | HJ_4 |

* = $^{32}$P labelled strand
