# Supplementary material for: The Human Homolog of Escherichia coli Endonuclease V Is a Nucleolar Protein with Affinity for Branched DNA Structures
Source: PLoS One. 2012 Nov 5;7(11):e47466. doi: 10.1371/journal.pone.0047466 (PMC3489907; doi:10.1371/journal.pone.0047466)
Supplement: Table S3 — Evaluation of the evolutionary selective pressure acting on ENDOV. (PDF) [file pone.0047466.s007.pdf]

Table S3. Evaluation of the evolutionary selective pressure acting on ENDOV. Ratio of amino acid replacing (*K*a) and silent (*K*s) mutation rates for pairwise comparisons of the endonuclease V core regions and C-terminal tails from mouse (GenBank [1] identifier NM_001164636), rat (Ensembl [2] transcript ENSRNOT00000054986) and hamster (XM_003496871) *ENDOV*. These species were selected for analysis because the mRNAs are processed by identical splicing of the same nine exons in the three rodents, simplifying analysis. *K*s is the number of synonymous substitutions per synonymous site and *K*a is the number of amino acid changing substitutions per non-synonymous site [3]. The calculation of the *K*a/*K*s ratio is a powerful tool for evaluating evolutionary selective pressure, with *K*a/*K*s < 1 indicating purifying (negative) selection, while *K*a/*K*s ~ 1 is indicating neutral evolution of the protein segment.

	*K*a/*K*s[a]	
	Mm ENDOV core	*Rn* ENDOV core
Rn ENDOV core	0.129 (0.123)	
Cg ENDOV core	0.233 (0.230)	0.214 (0.210)
	*K*a/*K*s[b]	
	Mm ENDOV C-term	*Rn* ENDOV C-term
Rn ENDOV C-term	1.424 (1.457)	
Cg ENDOV C-term	1.087 (1.137)	1.752 (1.880)

[a]*K*a/*K*s ratios for pairwise comparisons of ENDOV from mouse (*Mm*), rat (*Rn*), and Chinese hamster (*Cg*) for the core region (residues 1-250) calculated according to the model of Goldman and Yang [4] and the model averaging method of Zhang *et al.* (in parenthesis) [5] with the KaKs_Calculator [6].
[b]*K*a/*K*s ratios for pairwise comparisons of the C-terminal segments (codon 251 – stop) of ENDOV for the three rodents calculated as described above. Sites corresponding to insertions/deletions were removed.

Supporting Table S3 Reference List

1. Benson DA, Karsch-Mizrachi I, Clark K, Lipman DJ, Ostell J, and Sayers EW (2012) GenBank. Nucleic Acids Res 40: D48-D53.

2. Flicek P, Amode MR, Barrell D, Beal K, Brent S, *et al.* (2012) Ensembl 2012. Nucleic Acids Res 40: D84-D90.

3. Hurst LD (2002) The *K*a/*K*s ratio: diagnosing the form of sequence evolution. Trends Genet 18: 486-487.

4. Goldman N and Yang Z (1994) A codon-based model of nucleotide substitution for protein-coding DNA sequences. Mol Biol Evol 11: 725-736.

5. Zhang Z, Li J, Zhao XQ, Wang J, Wong GK, Yu J (2006) KaKs_Calculator: calculating *K*a and *K*s through model selection and model averaging. Genomics Proteomics Bioinformatics 4: 259-263.

6. Wang D, Zhang Y, Zhang Z, Zhu J, Yu J (2010) KaKs_Calculator 2.0: a toolkit incorporating gamma-series methods and sliding window strategies. Genomics Proteomics Bioinformatics 8: 77-80.
